# Age-Related Aesthetic Outcomes of Anterior Direct Composite Restorations: Color Match, Patient–Clinician Concordance, and Oral Health-Related Quality of Life

**DOI:** 10.3390/jcm15124610

**Published:** 2026-06-13

**Authors:** Magda Mihaela Luca, Roxana Buzatu, Bogdan Andrei Bumbu

**Affiliations:** 1 Department of Pediatric Dentistry, Faculty of Dental Medicine, “Victor Babes” University of Medicine and Pharmacy Timisoara, Eftimie Murgu Square 2, 300041 Timisoara, Romania; luca.magda@umft.ro; 2 Department of Dental Aesthetics, Faculty of Dental Medicine, “Victor Babes” University of Medicine and Pharmacy Timisoara, Revolutiei Boulevard 9, 300041 Timisoara, Romania; 3 Department of Dental Medicine, Faculty of Medicine and Pharmacy, University of Oradea, 410073 Oradea, Romania; bogdanbumbu@uoradea.ro

**Keywords:** dental esthetics, composite resins, color perception, patient satisfaction, quality of life

## Abstract

**Background/Objectives**: Anterior direct composite restorations are evaluated through instrumental color matching, clinician appraisal, and patient perception, but these endpoints may diverge by age. This cross-sectional study compared adolescents/young adults (AYA, 15–25 years) with adults/elderly (AE, 50–75 years) for spectrophotometric color difference (ΔE*ab), patient and clinician aesthetic ratings, patient–clinician agreement, and oral-health-related quality of life (OHRQoL). **Methods**: Consecutive recall patients with at least one anterior direct composite restoration placed ≥6 months earlier were screened; 128 were enrolled, and 126 completed all assessments (AYA n = 64; AE n = 62). Participants completed the OHIP-14 and aesthetic visual analogue scale (VAS) before receiving any USPHS, clinician VAS, or spectrophotometric feedback. A separate clinician, masked to patient scores and spectrophotometric outputs but not to patient age, recorded clinician VAS and modified USPHS criteria. **Results**: AE restorations showed higher ΔE*ab than AYA restorations (4.8 ± 1.6 vs. 3.2 ± 1.1; *p* < 0.001), whereas AYA reported lower patient VAS (72.4 ± 12.3 vs. 81.6 ± 10.8; *p* < 0.001) and higher OHIP-14 psychosocial burden (7.2 ± 2.8 vs. 4.0 ± 2.3; *p* < 0.001). Clinician VAS was higher in AYA (85.2 ± 7.3 vs. 79.4 ± 8.9; *p* < 0.001). Patient VAS correlated modestly with ΔE*ab (ρ = −0.38 in AYA; ρ = −0.31 in AE) and more strongly with psychosocial OHIP-14 scores (ρ = −0.54 and −0.47, respectively). Patient-clinician agreement was poor in AYA (ICC = 0.26) and moderate in AE (ICC = 0.58), with larger negative patient-minus-clinician discrepancies in AYA. Exploratory mediation statistically decomposed the age-related patient-satisfaction difference more through patient–clinician discrepancy than through ΔE*ab; causality cannot be inferred. **Conclusions**: Younger patients may experience dissatisfaction and psychosocial burden despite better instrumental color match. Assessment of anterior composites should combine objective shade measurement with patient-centered expectation clarification, and longitudinal studies should test temporal mechanisms and communication interventions.

## 1. Introduction

Dental aesthetics has shifted from a peripheral concern to a central element of contemporary restorative practice, propelled by patient expectations, greater visibility of dental appearance in everyday and digital social contexts, and improved material performance [1,2]. Anterior direct composite restorations now account for a growing share of aesthetic interventions across all age groups because they preserve sound tissue, are cost-effective, and allow single-visit delivery [3,4]. However, the clinical success of anterior composites is not defined by longevity alone: the restoration must blend optically with the surrounding dentition, resist extrinsic staining, and reproduce subtle value, chroma, gloss, surface texture, and translucency gradients that the human eye readily detects. These perceptual demands are especially consequential in the anterior zone, where even a single shade mismatch may undermine patient confidence despite formally acceptable clinical criteria.

Objective color matching is typically quantified by the CIELAB total color difference. A widely cited perceptibility threshold separates what an observer notices from what they overlook, while an acceptability threshold demarcates what clinicians and patients are willing to tolerate in the oral environment [5,6]. Achieving ΔE below these thresholds in vivo is non-trivial: layering technique, pre-polishing hydration, ambient lighting, and material brand each introduce variance [7]. More recent work has argued that the CIEDE2000 color-difference formula may better capture perceived differences at low ΔE levels, though CIELAB remains the operational standard in the majority of dental colorimetric cohorts [8]. Intraoral spectrophotometry, now increasingly available chair-side, provides operator-independent ΔE values and thereby enables a reproducible benchmark against which both patient-perceived and clinician-perceived outcomes can be compared in the same mouth.

Age introduces systematic shifts that challenge color matching. With advancing age, enamel thins, secondary and tertiary dentin accumulate, translucency decreases, and chroma in the yellow-red axis increases, producing teeth that are darker, more opaque, and more saturated than those of younger patients [9,10]. Parafunctional wear, extrinsic staining from tea, coffee, red wine, and tobacco, and pulpal recession further modify the optical target [11,12]. Consequently, the same shade-tab system that readily matches a 20-year-old central incisor may underperform when matching a 65-year-old tooth, a phenomenon that has been documented empirically but rarely quantified alongside patient-perceived aesthetic outcomes within the same cohort.

Beyond the physical restoration itself, dental aesthetics carries well-documented psychosocial weight and affects oral health-related quality of life (OHRQoL). Although aesthetics-specific instruments such as the Psychosocial Impact of Dental Aesthetics Questionnaire [13] offer granular coverage of appearance-related concerns, they have to date been validated in Romanian only in youth and young-adult samples [14], leaving a measurement gap for older Romanian populations that the present study was designed to address. The 14-item Oral Health Impact Profile (OHIP-14) is the most widely used generic OHRQoL instrument in dentistry, yielding a total score and seven two-item domains—functional limitation, physical pain, psychological discomfort, physical disability, psychological disability, social disability, and handicap. Its Romanian-language version has undergone formal translation, back-translation, and psychometric validation in a Romanian adult sample, with Cronbach’s α = 0.88, and demonstrated construct validity against clinical indices and has since been applied across the age spectrum in Romania, including in institutionalized elderly cohorts [15,16,17,18,19,20,21].

A further layer concerns discordance between clinician and patient evaluations of the same restoration. Clinicians usually judge color integration together with marginal adaptation, polish, surface texture, anatomy, and restoration integrity, whereas patients may focus more on visible brightness, symmetry, shade, and appearance during speech or photographs. When these reference frames differ, satisfaction can remain low even when conventional clinical criteria are acceptable. Previous work has described patient–clinician divergence in dental aesthetic assessment [22,23,24,25], but comparative age-stratified estimates using the same spectrophotometric, clinical, and patient-reported protocol remain limited.

The present study therefore compared two intentionally separated age strata: adolescents/young adults (15–25 years) and adults/elderly (50–75 years) with at least one anterior direct composite restoration placed at least 6 months previously. The 26–49-year interval was not sampled by design, because the objective was to maximize contrast between a developmentally younger group and a dentally aging group; including a transitional middle-adult group would have reduced the contrast needed for this exploratory age-comparison design. The objectives were: (i) to compare spectrophotometric color match and modified USPHS clinical quality by age group; (ii) to compare patient-rated and clinician-rated aesthetic outcomes, including absolute and signed disagreement; and (iii) to evaluate psychosocial burden and exploratory statistical decomposition of age-related differences through ΔE*ab or patient–clinician discrepancy. The a priori null hypothesis was that, after adjustment for measured clinical and behavioral factors, AYA and AE participants would not differ in ΔE*ab, patient aesthetic VAS, patient–clinician discrepancy, or OHIP-14 psychosocial subscale scores.

## 2. Materials and Methods

### 2.1. Study Design, Setting, and Participants

This was a cross-sectional, single-visit study conducted in the Department of Dental Aesthetics and the Department of Pediatric Dentistry at the Faculty of Dental Medicine, “Victor Babes” University of Medicine and Pharmacy Timisoara, between January 2024 and February 2025. All procedures adhered to the Declaration of Helsinki and the EU General Data Protection Regulation, and the protocol was approved by the Institutional Review Board (protocol code E-812, 12 December 2023). Written informed consent was obtained from all participants aged ≥18 years; for those aged 15–17 years, signed consent was obtained from a parent or legal guardian in addition to assent from the minor. Records were anonymized at extraction, secured on an encrypted institutional server, and accessed only by the study team through identifier codes. Reporting followed the STROBE statement for cross-sectional observational research [16].

Consecutive recall patients were screened over 13 months. Eligibility required: (i) age 15–25 years for the AYA stratum or 50–75 years for the AE stratum; (ii) at least one anterior direct resin-composite restoration on an incisor or canine placed ≥6 months before the visit; (iii) no systemic condition or medication known to alter saliva, mucosal health, or dental color; (iv) no extrinsic bleaching during the previous 6 months; and (v) no active caries on the restored tooth or directly adjacent teeth. The participant flow was reconciled as follows: 141 patients were screened, 13 were excluded before enrolment (no eligible anterior composite restoration, n = 5; restoration age < 6 months, n = 3; recent bleaching, n = 2; active caries on the index or adjacent tooth, n = 2; refusal after study information, n = 1), and 128 were enrolled. Two enrolled AE participants withdrew after baseline assessment because they could not complete the single-visit protocol; they did not contribute outcome data. The final complete-case sample was therefore 126 participants (64 AYA and 62 AE). The refusal rate among otherwise eligible patients was 1/127 (0.8%). A-priori sample-size estimation, targeting a medium between-group effect on the OHIP-14 psychosocial subscale (Cohen’s d = 0.5, two-sided α = 0.05, power = 0.80) [17], indicated a minimum of 64 participants per arm.

### 2.2. Clinical Examination and Spectrophotometric Assessment

Clinical and instrumental procedures were performed in the morning under standardized conditions in a dedicated operatory (D65-equivalent overhead illumination, ambient 22 ± 1 °C, relative humidity approximately 50%). Participants were asked to avoid eating, drinking anything except water, tooth-brushing, and mouthrinse use for at least 2 h before the visit. The index and reference teeth were polished with pumice using a rubber cup, rinsed for 60 s, and gently dabbed with sterile gauze. To reduce dehydration artifact, color measurements were obtained within 5 min after polishing/rinsing and before any prolonged photography, impressions, or shade discussion. The restored tooth and the homologous natural tooth were measured in the same short sequence. Two calibrated examiners independently recorded modified United States Public Health Service (USPHS/Ryge) criteria [19] for color match, surface texture, marginal adaptation, and marginal staining, using Alfa, Bravo, and Charlie categories. Inter-examiner reliability, assessed on 20 restorations before study initiation, was weighted κ ≥ 0.78 for all USPHS criteria.

Spectrophotometric color was recorded with a calibrated intraoral dental spectrophotometer (VITA Easyshade V, VITA Zahnfabrik, Bad Säckingen, Germany) after white-tile recalibration before each participant [18]. The probe was positioned perpendicular to the labial surface, with full tip contact and without visible marginal light leakage; unstable software readings or visibly incomplete seating led to immediate repeat measurement. Three sequential readings were taken at the middle third of the restoration and at the symmetric middle-third region of the adjacent homologous natural tooth. The exported mean of each triplicate was used for analysis to reduce random measurement error. Because the device export retained the averaged values rather than all individual replicate readings, a post-hoc intra-session ICC or coefficient of variation could not be calculated; this is now stated explicitly and treated as a measurement limitation. CIELAB coordinates (L*, a*, b*) were recorded, and ΔE*ab was calculated as [(ΔL*)^2^ + (Δa*)^2^ + (Δb*)^2^]^0.5^. In participants with multiple anterior restorations, the index restoration was selected using a fixed hierarchy: maxillary central incisor, then maxillary lateral incisor, then canine. If two restorations were at the same hierarchy level, the restoration with the larger visible restored surface area was selected; if still tied, the right-sided restoration was selected by convention before outcome analysis to avoid subjective post hoc choice. After patient-reported measures were completed, a second color-calibrated clinician, masked to patient scores and spectrophotometric output but necessarily aware of patient age, rated the index restoration on a 100 mm VAS anchored at 0 = ‘very unsatisfactory’ and 100 = ‘excellent, indistinguishable from natural tooth’.

### 2.3. Oral Health-Related Quality of Life, Psychosocial Subscale, and Covariates

Oral health-related quality of life and psychosocial impact were assessed with the Romanian-validated short-form Oral Health Impact Profile (OHIP-14) [15]. The OHIP-14 includes 14 items scored from 0 (never) to 4 (very often), producing a 0–56 total score and seven two-item domains: functional limitation, physical pain, psychological discomfort, physical disability, psychological disability, social disability, and handicap. The Romanian version was developed through forward-backward translation, pilot testing, and psychometric validation in Romanian adults, with Cronbach’s α = 0.88 and construct validity against self-perceived oral health and clinical indices [15]. We used OHIP-14 rather than an aesthetics-specific instrument such as PIDAQ [13], because Romanian PIDAQ validation is currently limited to youth and young-adult samples [14], whereas the present study required the same measurement framework across ages 15–75 years.

Within this validated OHIP-14 framework, the psychosocial endpoint was defined a priori as the sum of the three psychosocially relevant OHIP domains: Psychological Discomfort (items 5–6), Psychological Disability (items 9–10), and Social Disability (items 11–12), yielding a 0–24 score in which higher values indicate greater psychosocial burden. This six-item composite was pre-specified before analysis and was used as a domain-based summary rather than as a newly validated standalone questionnaire. Internal consistency in the present sample was acceptable (Cronbach’s α = 0.84 overall; α = 0.82 in AYA; α = 0.79 in AE). A threshold of ≥6, equivalent to an average domain-item response of at least ‘hardly ever,’ was retained as the operational endpoint for receiver-operating-characteristic analyses. Participants completed the OHIP-14 and patient aesthetic VAS in a quiet room without clinic staff present. The order of OHIP-14 versus VAS was alternated 1:1 across participants; however, both instruments were always completed before patients received any USPHS rating, clinician VAS result, or spectrophotometric output. Thus, patient VAS was not administered after clinical feedback and could not have been biased by disclosure of the clinician’s assessment. Behavioral and lifestyle covariates—daily tooth-brushing frequency, mouthrinse use, smoking status, daily coffee or black-tea consumption, and professional dental-visit frequency—were captured through a structured interviewer-administered questionnaire. Red wine, highly pigmented foods, detailed social-media exposure, dysmorphia symptoms, self-esteem, previous bleaching beyond 6 months, and prior aesthetic-treatment history were not captured and are treated as unmeasured covariates. Baseline DMFT index was recorded clinically.

### 2.4. Statistical Analysis

Continuous variables were summarized as mean ± standard deviation when approximately normally distributed (verified with histograms, Q–Q plots, and the Shapiro–Wilk test) and as median (interquartile range) otherwise. Categorical variables were reported as counts and proportions. Between-group comparisons (AYA vs. AE) used Welch’s independent-samples *t*-test for normally distributed continuous variables, the Mann–Whitney U test for skewed variables, and the χ^2^ test (or Fisher’s exact test when expected cell counts were <5) for categorical variables. Effect sizes were reported as Cohen’s d for continuous comparisons and Cramér’s V for categorical associations. Bivariate relationships among color match (ΔE), patient VAS, clinician VAS, the OHIP-14 psychosocial subscale, and OHIP-14 total were screened with Spearman’s rank correlation. All tests were two-sided with α set at 0.05.

Multivariable linear regression estimated adjusted associations with patient aesthetic VAS as the dependent variable. Prespecified predictors were age group (AYA reference), ΔE*ab, clinician VAS, restoration class (III, IV, V), sex, smoking status, time since placement, brushing frequency, and baseline DMFT; variance inflation factors < 3 were considered acceptable. Patient–clinician concordance was evaluated with ICC(2,1) absolute agreement [21], Bland–Altman bias and 95% limits of agreement [20], and weighted Cohen’s κ for categorical USPHS color ratings. Primary comparisons were age-group differences in ΔE*ab, patient VAS, patient–clinician discrepancy, and OHIP-14 psychosocial subscale. Domain-level, subgroup, mediation, and ROC analyses were considered exploratory; Benjamini-Hochberg false-discovery-rate checks were used for related secondary *p*-value families. Mediation analysis used quasi-Bayesian approximation with 5000 Monte-Carlo draws [22] and was interpreted only as a statistical decomposition. ROC analyses used Youden’s J and DeLong tests [23]. For the fitted combined logistic predictor, discrimination was supplemented by calibration metrics: Brier score, calibration intercept, calibration slope, and Hosmer–Lemeshow test. Analyses were conducted in R 4.3 (packages: mediation [22], pROC [24], irr, boot), and Python 3.11.

## 3. Results

Of 141 screened recall patients, 13 were excluded before enrolment, and two enrolled AE participants withdrew, leaving 126 complete cases (AYA n = 64, AE n = 62). The main findings were an AE disadvantage in ΔE*ab, but an AYA disadvantage in patient VAS, psychosocial burden, and patient–clinician concordance (Table 1).

The groups were well separated by age and differed in expected behavioral and restorative characteristics. AE participants had higher DMFT, more smoking, more daily coffee/tea use, more anterior composites, and longer time since placement, while sex distribution was similar. These variables informed the adjusted models (Table 2).

AE restorations showed higher ΔE*ab than AYA (4.8 ± 1.6 vs. 3.2 ± 1.1; *p* < 0.001), mainly through lightness and yellow-blue differences. Acceptable color match (ΔE*ab ≤ 2.7), USPHS color Alfa scores, marginal-staining Alfa scores, and overall USPHS Alfa status were all less frequent in AE; surface texture and marginal adaptation did not differ significantly (Table 3).

AYA participants had higher psychosocial OHIP-14 burden than AE despite lower functional-limitation scores in AE. The pre-specified psychosocial subscale was higher in AYA (7.2 ± 2.8 vs. 4.0 ± 2.3; *p* < 0.001), and clinically meaningful psychosocial impact (≥6) was more common in AYA (60.9% vs. 22.6%), as seen in Table 4.

Patient VAS was lower in AYA than AE overall (72.4 ± 12.3 vs. 81.6 ± 10.8; *p* < 0.001) and within each restoration-class and tooth-position stratum. Patient VAS correlated modestly with ΔE*ab and more strongly with OHIP-14 psychosocial scores, supporting a perception-related component beyond objective color mismatch (Table 5).

Clinicians rated AYA restorations higher than AE restorations, but patients rated them lower. Signed discrepancy was negative in AYA and near-neutral in AE (−12.8 vs. +2.2 VAS points; *p* < 0.001), and absolute disagreement > 10 points was more frequent in AYA (57.8% vs. 27.4%). The USPHS color agreement was also lower in AYA (Table 6).

**Figure 1:**
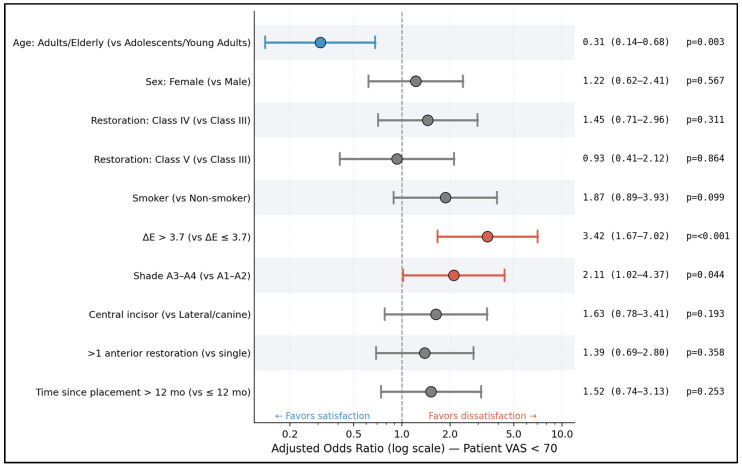
Adjusted odds ratios for aesthetic dissatisfaction (patient VAS < 70).

In adjusted regression, AE status, lower ΔE*ab, and higher clinician VAS were independently associated with higher patient VAS. Restoration class, sex, smoking, time since placement, brushing, and DMFT were not significant after adjustment. The model explained 47% of patient–VAS variance (Figure 1).

**Figure 2:**
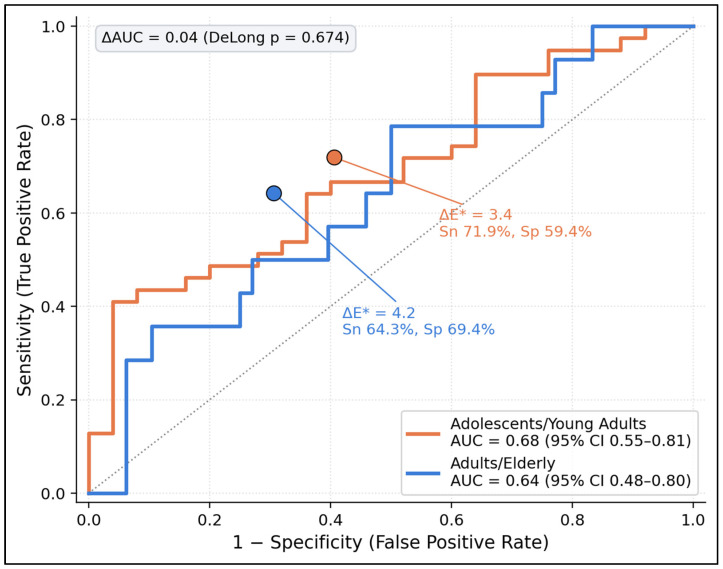
ROC curves for ΔE*ab predicting clinically meaningful OHIP-14 psychosocial impact (subscale ≥ 6) by age group.

AE status was associated with lower dissatisfaction odds, while ΔE*ab > 3.7 and darker baseline shade were associated with higher odds. Other variables were not significant; results should be interpreted as adjusted associations, not causal effects (Figure 2).

**Figure 3:**
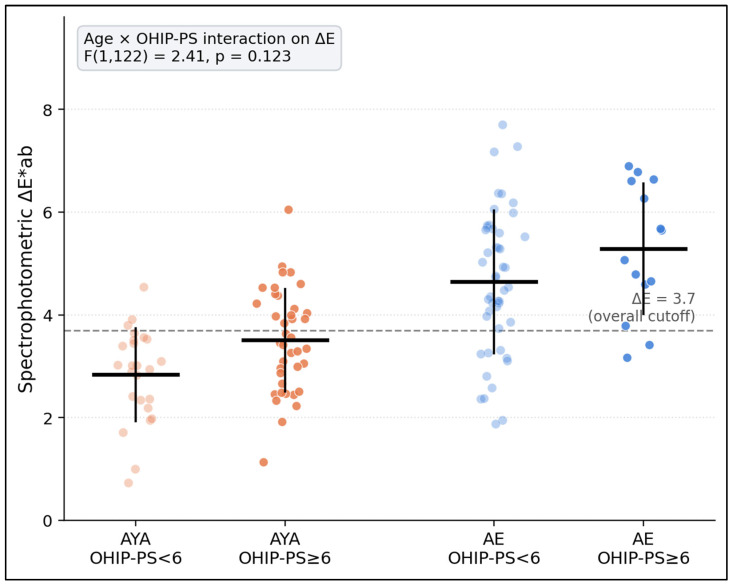
Jittered individual-value plot of ΔE*ab by age group and psychosocial-impact category. Dots represent individual restorations; horizontal bars show the subgroup mean, and vertical whiskers show ±1 standard deviation. The figure was retained as an individual-value display rather than converted to a box plot because the subgroup sample sizes are modest and the individual observations are informative.

ΔE*ab discrimination was modest in both age groups (AUC 0.68 in AYA; 0.64 in AE), with no significant between-stratum AUC difference (*p* = 0.674). Age-specific cutoffs were exploratory only (Figure 3).

ΔE*ab values were generally higher in AE than in AYA, whereas psychosocial impact was more frequent in AYA. Observations with ΔE*ab > 7 were retained after chart review; sensitivity checks excluding these observations did not change the main age-group associations (Table 7).

Agreement metrics consistently showed weaker patient-clinician concordance in AYA than AE. AYA had a lower ICC, a larger negative Bland–Altman bias, a significant proportional bias, lower weighted κ, and fewer ratings within ±5 VAS points of the clinician (Table 8).

Mediation models were exploratory only. The age association with patient VAS was statistically decomposed more through patient–clinician discrepancy than through ΔE*ab, but reverse causality and unmeasured mediator–outcome confounding remain possible (Table 9).

For OHIP-14 psychosocial impact ≥ 6, ΔE*ab showed modest discrimination, absolute patient–clinician discrepancy performed better, and the combined model had the highest AUC. Calibration was acceptable in this dataset, but all ROC cutoffs require external validation.

## 4. Discussion

### 4.1. Literature Findings

Our cross-sectional comparison showed an age-related aesthetic paradox: AYA participants had better instrumental color match and higher clinician aesthetic ratings, yet they reported lower satisfaction and greater psychosocial burden than AE participants [25,26,27,28]. These results reject the null hypothesis of no age-related difference in objective, perceptual, and psychosocial outcomes. They should not be read as evidence that AYA restorations were clinically inferior. Instead, they support the interpretation that technical success and patient-valued success are related but distinct constructs, consistent with patient-reported outcome literature in dentistry [29,30,31,32,33].

A stricter aesthetic threshold in younger patients is one possible explanation, but this remains an untested hypothesis in the present dataset. We did not measure social-media exposure, peer comparison, self-esteem, body-image concerns, or dysmorphia symptoms; therefore, the study cannot attribute the AYA dissatisfaction pattern to any specific psychosocial mechanism. The more cautious interpretation is that AYA participants showed greater expectation or perception discordance. Future studies should measure these candidate mechanisms prospectively and should combine generic OHRQoL tools with validated aesthetics-specific instruments [34,35,36,37].

The mediation analysis provides a structured description of associations but not a causal pathway. The larger indirect component through patient–clinician discrepancy is compatible with a perceptual-discordance explanation; however, the opposite direction is also plausible, because patients with greater pre-existing psychosocial burden may rate restorations more critically and thereby create larger discrepancies. Longitudinal studies that measure pretreatment expectations, immediate post-placement satisfaction, and later color change are required before temporality can be established [38,39,40]. This caution is reinforced by the use of OHIP-14, a generic OHRQoL instrument. Although it permits cross-age comparison, it may be less sensitive to appearance-specific concerns than PIDAQ or similar tools, and such instruments would require validation across the full Romanian age range before replacing OHIP-14 in comparative age research.

Clinically, these results support a dual assessment approach rather than reliance on color metrics alone. Instrumental shade measurement can document optical mismatch, while structured expectation-setting can clarify which features matter most to the patient, such as shade, translucency, shape, brightness, and appearance in photographs. Communication tools proposed in esthetic dentistry, including shared standardized photographs and digital smile-design planning, may help align expectations [30]; however, the present study did not test such interventions, and controlled trials in AYA patients are still needed. Residual confounding from unmeasured patient, operator, and treatment factors also remains possible [41,42,43,44,45,46,47,48,49].

### 4.2. Study Limitations

Several limitations merit explicit consideration. First, the cross-sectional single-center design cannot establish causality. Patient–clinician discrepancy may contribute to dissatisfaction, but reverse causality is also plausible: participants with greater pre-existing psychosocial burden could rate restorations more critically and thereby generate larger discrepancies. The mediation models, therefore, represent exploratory statistical decomposition only. Second, the Romanian single-center recall sample limits external validity. Cultural norms, socioeconomic position, prior exposure to restorative dentistry, and age-related response shift may affect aesthetic expectations and tolerance thresholds [50,51]. Consecutive recall recruitment reduced overt sampling discretion, but it may have selected more compliant patients with better dental attendance; the observed refusal rate was low, but the results may not generalize to less compliant or treatment-seeking populations.

Third, several potentially important confounders were not measured. We did not quantify social-media exposure, self-esteem, body-image concerns, dysmorphia symptoms, detailed red-wine or pigmented-food intake, bleaching history beyond 6 months, or prior aesthetic-treatment expectations. Coffee/tea and smoking were recorded, but pigment exposure remains incompletely captured, particularly because AE restorations had a slightly longer mean time since placement. Original operator identity, operator gender, specialty level, and exact restorative protocol at placement were also unavailable; consequently, operator-selection effects cannot be excluded. Because restorations were evaluated in a controlled academic setting, findings should not be extrapolated to all community-practice operators without validation.

Fourth, measurement limitations should be emphasized. OHIP-14 was selected to maintain Romanian-language measurement equivalence across ages 15–75 years, but it is a generic OHRQoL instrument rather than an aesthetics-specific scale. Differential item functioning across age groups was not tested; if individual OHIP-14 items are interpreted differently by younger and older participants, between-group comparisons may be biased even when the same validated instrument is used [52,53]. Future work should evaluate measurement invariance and, ideally, validate aesthetics-specific instruments for older Romanian adults. Spectrophotometric assessment also has limitations: ΔE*ab was measured only at the middle third and cannot fully represent incisal translucency, polychromatic layering, gloss, surface texture, or edge effects on curved enamel. Probe seating was standardized, but age-related enamel morphology could still influence readings. In addition, the analysis dataset retained triplicate means rather than all replicate measurements, so a formal post-hoc intra-session ICC or coefficient of variation could not be reported. CIEDE2000 was not calculated, and future studies should report both ΔE*ab and ΔE00. Finally, subgroup and ROC analyses were underpowered for definitive class-specific or age-specific thresholds, and the cutoffs should be treated as exploratory.

## 5. Conclusions

In this cross-sectional single-center cohort, age group was associated with different aesthetic and psychosocial profiles after anterior direct composite restorations. Younger patients showed better objective color match but greater patient–clinician discordance and psychosocial burden, indicating that technical color success alone does not fully predict patient-perceived success. The findings support a dual patient-centered assessment strategy that combines instrumental color evaluation with explicit expectation-setting and shared review of aesthetic goals. Because the mediation and threshold analyses are exploratory, longitudinal, and multi-center studies using both OHIP-14 and validated aesthetics-specific instruments are needed to confirm temporal pathways, improve measurement invariance, and test whether structured communication reduces psychosocial burden.

## Figures and Tables

**Table 1:** Baseline demographic, behavioral, and restoration characteristics by age group.

Variable	AYA (n = 64)	AE (n = 62)	*p*-Value	Effect Size
Age (years, mean ± SD)	20.3 ± 3.2	61.7 ± 7.4	<0.001 ^1^	d = 7.31
Female, n (%)	35 (54.7)	36 (58.1)	0.702 ^2^	V = 0.034
Education > secondary, n (%)	41 (64.1)	27 (43.5)	0.021 ^2^	V = 0.207
Current smoker, n (%)	8 (12.5)	21 (33.9)	0.004 ^2^	V = 0.257
Daily coffee/tea, n (%)	36 (56.3)	49 (79.0)	0.007 ^2^	V = 0.241
Brushing (times/day, mean ± SD)	2.1 ± 0.5	1.9 ± 0.6	0.043 ^1^	d = 0.36
Baseline DMFT (mean ± SD)	3.2 ± 1.6	8.7 ± 2.9	<0.001 ^1^	d = 2.33
No. anterior composites (mean ± SD)	1.8 ± 0.9	2.3 ± 1.1	0.007 ^1^	d = 0.49
Time since placement (months, mean ± SD)	9.4 ± 3.1	11.2 ± 4.3	0.008 ^1^	d = 0.48
Index restoration class III, n (%)	28 (43.8)	22 (35.5)		
Index restoration class IV, n (%)	24 (37.5)	19 (30.6)	0.114 ^2^	V = 0.178
Index restoration class V, n (%)	12 (18.8)	21 (33.9)		
Index tooth: central incisor, n (%)	33 (51.6)	26 (41.9)	0.279 ^2^	V = 0.097

^1^ Welch’s independent-samples *t*-test; ^2^ χ^2^ test. AYA = adolescents/young adults; AE = adults/elderly; DMFT = decayed-missing-filled teeth; d = Cohen’s d; V = Cramér’s V.

**Table 2:** Spectrophotometric color match and modified USPHS clinical ratings at the index restoration.

Parameter	AYA (n = 64)	AE (n = 62)	*p*-Value	Effect Size
ΔE*ab (CIE 1976)	3.2 ± 1.1	4.8 ± 1.6	<0.001 ^1^	d = 1.16
ΔL* (lightness difference)	−0.8 ± 1.3	−2.1 ± 1.7	<0.001 ^1^	d = 0.86
Δa* (red–green axis)	0.3 ± 0.6	0.7 ± 0.9	0.004 ^1^	d = 0.52
Δb* (yellow–blue axis)	1.1 ± 1.2	2.3 ± 1.6	<0.001 ^1^	d = 0.85
ΔE within acceptability (≤2.7), n (%)	41 (64.1)	18 (29.0)	<0.001 ^2^	V = 0.353
Color match: Alfa, n (%)	41 (64.1)	28 (45.2)	0.033 ^2^	V = 0.190
Surface texture: Alfa, n (%)	48 (75.0)	39 (62.9)	0.142 ^2^	V = 0.131
Marginal staining: Alfa, n (%)	52 (81.3)	36 (58.1)	0.004 ^2^	V = 0.256
Marginal adaptation: Alfa, n (%)	51 (79.7)	45 (72.6)	0.346 ^2^	V = 0.083
Overall USPHS Alfa across all 4 criteria, n (%)	29 (45.3)	14 (22.6)	0.007 ^2^	V = 0.241

^1^ Welch’s *t*-test; ^2^ χ^2^ test. ΔE*ab computed per CIE 1976 formula between the restoration mid-third and the homologous natural tooth mid-third. Acceptability threshold = 2.7. CIEDE2000 was not calculated because the clinical protocol and instrument export were based on CIELAB; consequently, all color-difference interpretations refer specifically to ΔE*ab.

**Table 3:** OHIP-14 domain scores, total, and pre-specified psychosocial subscale by age group.

OHIP-14 Measure (Range)	AYA (n = 64)	AE (n = 62)	*p*-Value	Cohen’s d
Functional limitation (0–8)	1.9 ± 0.9	2.6 ± 1.2	<0.001 ^1^	0.66
Physical pain (0–8)	1.4 ± 0.8	1.7 ± 1.0	0.068 ^1^	0.33
Psychological discomfort (0–8) †	3.1 ± 1.3	1.8 ± 1.1	<0.001 ^1^	1.08
Physical disability (0–8)	1.9 ± 0.9	1.8 ± 1.0	0.548 ^1^	0.11
Psychological disability (0–8) †	2.4 ± 1.1	1.3 ± 0.9	<0.001 ^1^	1.09
Social disability (0–8) †	1.7 ± 0.9	0.9 ± 0.7	<0.001 ^1^	0.99
Handicap (0–8)	0.8 ± 0.6	0.5 ± 0.5	0.003 ^1^	0.54
OHIP-14 total (0–56)	13.2 ± 4.1	10.7 ± 3.8	<0.001 ^1^	0.63
Psychosocial subscale (0–24, PD + PSD + SD) †	7.2 ± 2.8	4.0 ± 2.3	<0.001 ^1^	1.25
Psychosocial subscale ≥ 6 (clinically relevant), n (%)	39 (60.9)	14 (22.6)	<0.001 ^2^	V = 0.388

^1^ Welch’s *t*-test; ^2^ χ^2^ test. † Components of the pre-specified psychosocial subscale (Psychological Discomfort [PD] + Psychological Disability [PSD] + Social Disability [SD]; range 0–24, higher = greater psychosocial burden). The Romanian-validated version of OHIP-14 was used throughout. Multiplicity note: domain-level OHIP-14 comparisons were considered exploratory. After Benjamini-Hochberg false-discovery-rate sensitivity checking within the OHIP-14 family, psychological discomfort, psychological disability, social disability, handicap, OHIP-14 total, and the psychosocial subscale remained significant, whereas physical pain and physical disability remained non-significant.

**Table 4:** Patient aesthetic satisfaction (VAS) by age group, overall, and stratified by restoration and tooth characteristics.

Stratum	AYA (n = 64)	AE (n = 62)	*p*-Value	Cohen’s d
Overall cohort				
Patient aesthetic VAS (0–100)	72.4 ± 12.3	81.6 ± 10.8	<0.001 ^1^	0.79
Patient VAS < 70 (dissatisfied), n (%)	34 (53.1)	19 (30.6)	0.011 ^2^	V = 0.230
Stratified by restoration class				
Class III (proximal surface)	74.1 ± 11.2	82.9 ± 10.1	<0.001 ^1^	0.82
Class IV (incisal edge/angle)	68.2 ± 13.1	79.3 ± 11.2	<0.001 ^1^	0.91
Class V (cervical)	73.1 ± 12.8	82.7 ± 9.4	0.006 ^1^	0.83
Stratified by index tooth type				
Maxillary central incisor	70.9 ± 11.7	80.4 ± 10.3	<0.001 ^1^	0.86
Lateral incisor or canine	74.2 ± 12.9	82.8 ± 11.1	0.002 ^1^	0.71
Correlation with objective measures				
Patient VAS vs. ΔE*ab (Spearman ρ)	−0.38, *p* = 0.002 ^3^	−0.31, *p* = 0.014 ^3^	—	—
Patient VAS vs. OHIP-14 psychosocial (Spearman ρ)	−0.54, *p* < 0.001 ^3^	−0.47, *p* < 0.001 ^3^	—	—

^1^ Welch’s *t*-test; ^2^ χ^2^ test; ^3^ Spearman rank correlation (two-sided). VAS = visual analogue scale.

**Table 5:** Patient versus clinician aesthetic ratings and their discrepancies.

Measure	AYA (n = 64)	AE (n = 62)	*p*-Value	Effect Size
Clinician aesthetic VAS (0–100)	85.2 ± 7.3	79.4 ± 8.9	<0.001 ^1^	d = 0.71
Patient aesthetic VAS (0–100)	72.4 ± 12.3	81.6 ± 10.8	<0.001 ^1^	d = 0.79
Signed discrepancy (Patient − Clinician)	−12.8 ± 9.7	+2.2 ± 8.1	<0.001 ^1^	d = 1.68
Absolute discrepancy |Patient − Clinician|	13.4 ± 8.9	7.1 ± 5.3	<0.001 ^1^	d = 0.86
Absolute discrepancy > 10 points, n (%)	37 (57.8)	17 (27.4)	<0.001 ^2^	V = 0.310
Patient rated lower than clinician, n (%)	48 (75.0)	24 (38.7)	<0.001 ^2^	V = 0.367
Categorical USPHS color match agreement, % (n)	67.2 (43/64)	80.6 (50/62)	0.085 ^2^	V = 0.151
Weighted κ (patient vs. clinician, USPHS color)	0.31 (0.17–0.45)	0.52 (0.38–0.66)	— ^3^	— ^3^

^1^ Welch’s *t*-test; ^2^ χ^2^ test; ^3^ 95% CIs by bootstrap (2000 resamples); between-stratum κ comparison via Donner–Eliasziw Z-test, *p* = 0.027.

**Table 6:** Multivariable linear regression for patient aesthetic satisfaction (Patient VAS).

Predictor	β (95% CI)	Std. β	*p*-Value	VIF
Intercept	68.4 (58.9 to 77.9)	—	<0.001	—
Age group: AE (vs. AYA reference)	+7.2 (+3.8 to +10.6)	+0.31	<0.001	1.46
ΔE*ab (per 1 unit)	−2.3 (−3.1 to −1.5)	−0.28	<0.001	1.62
Clinician VAS (per 1 unit)	+0.24 (+0.13 to +0.35)	+0.21	<0.001	1.33
Restoration class IV (vs. III)	−1.9 (−5.2 to +1.4)	−0.07	0.259	1.24
Restoration class V (vs. III)	−0.7 (−4.6 to +3.2)	−0.03	0.723	1.29
Female sex	−1.1 (−4.3 to +2.1)	−0.05	0.497	1.11
Current smoker	−3.2 (−7.1 to +0.7)	−0.10	0.108	1.34
Time since placement (per month)	−0.11 (−0.47 to +0.25)	−0.04	0.548	1.38
Brushing frequency (per time/day)	+0.9 (−1.8 to +3.6)	+0.05	0.517	1.18
Baseline DMFT (per unit)	−0.2 (−0.7 to +0.3)	−0.04	0.428	1.74

Dependent variable: patient aesthetic VAS (0–100). Model R^2^ = 0.47; adjusted R^2^ = 0.43; F(10,115) = 10.2, *p* < 0.001. HC3 robust standard errors. VIF = variance inflation factor (all <3, no multicollinearity concern).

**Table 7:** Patient–clinician rating concordance and agreement metrics by age group.

Metric	AYA (n = 64)	AE (n = 62)	Overall (n = 126)	Between-Group Test
ICC(2,1), absolute agreement (95% CI)	0.26 (0.03–0.48)	0.58 (0.41–0.72)	0.42 (0.28–0.55)	Fisher z, *p* = 0.014
Pearson r (Patient vs. Clinician VAS)	0.34 (*p* = 0.006)	0.61 (*p* < 0.001)	0.51 (*p* < 0.001)	Fisher z, *p* = 0.041
Bland–Altman mean bias (VAS points)	−12.8	+2.2	−5.3	t = 9.43, *p* < 0.001
95% Limits of Agreement (LoA)	−31.8 to +6.2	−13.7 to +18.1	−28.4 to +17.8	—
Proportional bias (slope on mean VAS)	−0.18 (*p* = 0.027)	−0.04 (*p* = 0.521)	−0.11 (*p* = 0.031)	—
Weighted κ, USPHS color match	0.31 (0.17–0.45)	0.52 (0.38–0.66)	0.43 (0.32–0.54)	Donner–Eliasziw, *p* = 0.027
% within 5-VAS-point agreement	25.0 (16/64)	54.8 (34/62)	39.7 (50/126)	χ^2^, *p* < 0.001
% within 10-VAS-point agreement	42.2 (27/64)	72.6 (45/62)	57.1 (72/126)	χ^2^, *p* < 0.001

ICC = intraclass correlation coefficient, two-way mixed-effects, absolute agreement definition. Proportional bias tested by regressing patient–clinician difference on the average of the two ratings (significant negative slope indicates greater disagreement at higher rating means).

**Table 8:** Exploratory mediation analysis of the age-group association with the OHIP-14 psychosocial subscale and patient satisfaction (patient VAS).

Causal pathway	Estimate (95% CI)	*p*-Value	Proportion Mediated
Age group → OHIP-14 psychosocial subscale, mediated by ΔE*ab			
Total effect (AE vs. AYA)	−3.2 (−4.2 to −2.2)	<0.001	—
Average Direct Effect (ADE)	−2.5 (−3.5 to −1.5)	<0.001	—
Average Causal Mediation Effect (ACME)	−0.7 (−1.1 to −0.3)	0.004	20.4%
Age group → Patient VAS, mediated by patient–clinician discrepancy			
Total effect (AE vs. AYA)	+9.2 (+5.1 to +13.3)	<0.001	—
Average Direct Effect (ADE)	+3.1 (−0.6 to +6.8)	0.097	—
Average Causal Mediation Effect (ACME)	+6.1 (+4.3 to +7.9)	<0.001	66.3%
Age group → OHIP-14 psychosocial subscale, mediated by patient–clinician discrepancy			
Total effect (AE vs. AYA)	−3.2 (−4.2 to −2.2)	<0.001	—
Average Direct Effect (ADE)	−1.3 (−2.4 to −0.2)	0.017	—
Average Causal Mediation Effect (ACME)	−1.9 (−2.7 to −1.1)	<0.001	59.1%

Mediation via quasi-Bayesian approximation with 5000 Monte-Carlo draws (R package mediation). ACME = average causal mediation effect, the model-based indirect component through the specified mediator; ADE = average direct effect, the component not statistically passing through that mediator. These models assume correct temporal ordering and no unmeasured mediator-outcome confounding, assumptions that cannot be verified in this cross-sectional design. Sensitivity analysis via Imai’s ρ indicated that moderate unmeasured mediator-outcome confounding could attenuate the indirect effects; therefore, mediation estimates are reported as exploratory statistical decompositions rather than causal estimates.

**Table 9:** Discriminative performance of selected predictors for moderate-to-severe psychosocial impact (OHIP-14 psychosocial subscale ≥ 6).

Predictor	AUC (95% CI)	Optimal Cutoff	Sn (%)	Sp (%)	PPV (%)	NPV (%)
ΔE*ab (overall cohort, n = 126)	0.71 (0.62–0.80)	3.7	74.2	61.8	58.4	76.9
ΔE*ab—AYA subgroup	0.68 (0.55–0.81)	3.4	71.9	59.4	63.2	68.8
ΔE*ab—AE subgroup	0.64 (0.48–0.80)	4.2	64.3	69.4	37.3	87.5
|Patient − Clinician VAS| (overall)	0.78 (0.70–0.86)	9.5	76.7	74.8	68.2	82.1
|Patient − Clinician VAS|—AYA	0.74 (0.62–0.86)	11.2	72.2	67.9	72.2	67.9
|Patient − Clinician VAS|—AE	0.81 (0.68–0.94)	7.4	75.0	78.3	50.0	91.8
Clinician VAS (overall)	0.59 (0.49–0.69)	82.5	61.4	57.2	54.9	63.6
ΔE + |P–C discrepancy| (combined, logistic fit)	0.83 (0.75–0.90)	— ^1^	79.4	76.9	70.3	84.4

^1^ Composite linear predictor; no single cutoff exists on the original measurement scales. Sn = sensitivity; Sp = specificity; PPV = positive predictive value; NPV = negative predictive value. DeLong comparison: AUC (combined predictor) versus AUC (ΔE*ab overall) difference = 0.12, 95% CI 0.04–0.20, *p* = 0.003. Calibration was assessed for the fitted combined logistic probability model and was acceptable in this exploratory dataset (Brier score = 0.16; calibration intercept = 0.02; calibration slope = 0.91; Hosmer–Lemeshow *p* = 0.44). These calibration statistics do not apply in the same way to single-marker ROC cutoffs, which produce discrimination thresholds rather than full predicted probabilities.

## Data Availability

The data presented in this study are available on reasonable request from the corresponding author, subject to institutional approval.
